# An evaluation of a short film promoting kindness in Wales during COVID-19 restrictions #TimeToBeKind

**DOI:** 10.1186/s12889-022-12876-9

**Published:** 2022-03-24

**Authors:** Kat Ford, Mark A Bellis, Rebecca Hill, Karen Hughes

**Affiliations:** 1 Public Health Collaborating Unit, College of Human Sciences, Bangor University, LL13 7YP Wrexham, UK; 2 World Health Organization Collaborating Centre on Investment for Health and Well-being, Policy and International Health, Public Health Wales, LL13 7YP Wrexham, UK; 3 World Health Organization Collaborating Centre on Investment for Health and Well-being, Policy and International Health, Public Health Wales, CF10 4BZ Cardiff, UK

**Keywords:** Adverse childhood experiences, Public health, Health communication, Behaviour change, Kindness, COVID-19, Social media

## Abstract

**Background:**

In response to successive COVID-19 restrictions in Wales, the Welsh ACE Support Hub launched the #TimeToBeKind campaign in March 2021. The campaign used a short film broadcast on national television and promoted on social media to encourage behaviour change for kindness. We evaluated the #TimeToBeKind campaign film to identify whether watching the film would result in increased intention to act in ways that promote kindness to others and if intentions were associated with being emotionally affected by the film.

**Methods:**

A mixed methods evaluation was employed, using a short online survey and interaction with the film on the Twitter social media platform. The online survey measured public (*n* = 390) attitudes towards the film including feelings invoked, and behavioural intentions for acts of kindness as a result of viewing the film. Tweets which interacted with the film (*n* = 59; likes, re-tweets or comments), and tweet sentiment (positive, negative, or neutral) towards the film were also explored.

**Results:**

The majority of participants reported positive attitudes to the film and agreed that they understood the campaign message (91.8%). 67.9% reported that the film made them feel upset or sad and for 22.6% the film resonated with their lockdown experience. As a result of seeing the film, 63.6% reported intentions to be kinder to others, 65.6% intended to try and help other members of their community, and 70.5% were more likely to check in on friends, family and neighbours. A higher proportion of individuals who were emotionally affected by the film (e.g. upset or sad, hopeful or encouraged, gained something positive) and those for whom the film resonated with their lockdown experience reported increased kindness behavioural intentions as a result of seeing the film.

**Conclusions:**

Film can be an effective tool to promote behaviour change for kindness. Films that provoke strong emotional reactions can still be perceived positively and lead to behaviour change. With the COVID-19 pandemic accelerating a move online for many, the findings of the present evaluation are relevant to how public health messaging can adapt and utilise this space to target individuals and promote behaviour change.

## Background

COVID-19 is a global public health emergency with widespread impacts on families and children. National and regional restrictions put in place to manage the pandemic have limited face-to-face social interaction, employment opportunities, and access to education, health and support services. Bans on household mixing and socialisation have had negative consequences for population health and wellbeing and have been associated with increases in mental illness, low mental wellbeing and suicide [1, 2]. For children, limited access to support services and education may have academic, health, and economic consequences into adulthood. Critically, for some, lockdown within the home has led to increased exposure to domestic violence, child maltreatment and other adverse childhood experiences (ACEs; stressful experiences in childhood such as experiencing physical, sexual or emotional abuse, or growing up in a household with parental substance abuse or mental illness) [3, 4], along with delays in presentations of child protection cases [5, 6]. Further, there is concern that COVID-19 restrictions may have increased risk factors for ACEs, such as parental unemployment and stress [7–10]. Thus, despite reduced reports of violence to police and health services during the pandemic, there has been increased demand for third sector organisations providing support, particularly increased reporting of concern for potential ACE exposure [11]. A growing international evidence base identifies strong relationships between exposure to ACEs and the adoption of health-harming behaviours (e.g. smoking, binge drinking and illicit drug use), poor educational attainment and increased anti-social behaviour and violence involvement [12, 13]. Evidence also finds a link between the experience of ACEs and the development of poor physical health, chronic disease and mental illness [14, 15]. Exposure to ACEs is relatively common, with almost half of adults in Wales reporting the experience of at least one ACE, and 12.6% reporting exposure to four or more ACEs [16]. Thus, the prevention of ACEs and support for families affected by them is critical in improving population health and wellbeing and increasingly features within public health policy [17].

The risk of negative outcomes in those exposed to ACEs are reduced through resilience resources, particularly having a trusted adult or role model during childhood [18, 19]. At the same time as providing an increased risk of children and young people directly experiencing and observing abuse, COVID-19 restrictions have also limited sources of external support [5, 8, 20]. Globally, essential social networks and support structures have been closed, limiting activities which help to build resilience. Limited resilience resources as a result of the pandemic may have also been further exacerbated for those in poverty whose access to resources and physical space, including green space, may be even further restricted. Furthermore, resources for many services and programmes known to help prevent ACEs or build resilience (e.g. parenting programmes) have also been diverted due to the pandemic. In light of such changes to essential support structures, there is a need to understand how individuals can be supported beyond the scope of the professional response. With those exposed to ACEs already at increased risk for poor mental health and wellbeing [21], the effects of COVID-19 restrictions will disproportionally affect the disadvantaged [6].

In response to a sustained period of successive national and regional COVID-19 restrictions in Wales, in 2021 the Welsh ACE Support Hub, a Welsh Government funded collaborative organisation established to tackle ACEs, launched the #TimeToBeKind campaign. The ambition of the ACE Support Hub for the 2021 #TimeToBeKind campaign was to encourage local action or behaviour change for kindness. The campaign producers aimed to encourage individuals to think about how the pandemic has affected themselves and others and prompt them to be more compassionate to other members of society. However, the campaign did not provide a definition for kindness or compassion. The campaign built on a previous 2019 campaign, which received 2 million TV impressions and resulted in increased engagement with the ACE Support Hub social media accounts and website [22], but was not formally evaluated. The 2021 campaign ran for a six-week period (16th March-27th April 2021) and comprised a short film broadcast on national television in Wales (shown on S4C and ITV Wales channels 60 times during prime time programming, with over 2.2 million viewers across both channels seeing the advert at least once) [23]. Social media posts by the ACE Support Hub and their stakeholders used the hashtag #TimeToBeKind to encourage campaign engagement. Other campaign features were hosted on the campaign webpage (e.g. ‘Smile cinema’ and ‘Kindness map’) but were not included in this evaluation.

**Fig. 1:**
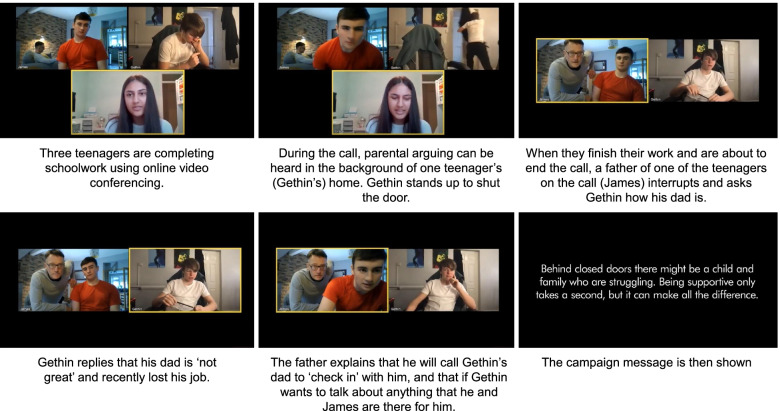
Storyboard of #TimeToBeKind campaign film

The 2021 campaign film is outlined in Fig. 1. The film depicts a scenario where three teenagers are completing schoolwork using online video conferencing. During the video call a parent reaches out to a young person on the call who is experiencing childhood adversity (parental arguing) at home. The film is 60 s long (a shorter 30 s version was also created), available in both English and Welsh languages (available with and without subtitles) and accessible on the YouTube platform (English, https://www.youtube.com/watch?v=B4Tk7Jg8mfE; Welsh, https://www.youtube.com/watch?v=0htJpP9KYhA).

Permission has been granted for the reproduction of screen shots from the #TimeToBeKind campaign film by the film publisher, The Welsh ACE Support Hub.

Within the field of public health, public campaigns have been commonly used to promote positive behaviour change such as seat belt use, healthy living, dental care and substance use prevention. Film and animation are increasingly used as a health promotion tool in such campaigns to facilitate behaviour change. Examples of this include for oral health promotion [24], public awareness of antibiotic use [25], ACEs [26], and communication on COVID-19 [27]. Research has identified that films that have a strong influence on behaviour change: provide clear and simple messages; convey their message in an accessible format to widespread audiences including those with low literacy, and underrepresented or difficult to reach communities; are credible; provoke viewer emotions; and contain positive empowering messages [28–31]. Campaigns which use online media have also been found to receive increased engagement and be cost-effective [32]. However, films promoting behaviour change are rarely evaluated. Increased use of online technology including social media has opened up a way of both promoting and evaluating public information films. Such methods provide a novel opportunity to explore the ability of films to reach individuals at home. Although the film evaluated here was created in response to COVID-19, it provides a method of dissemination that can be applied to broader public health information films that seek to target individuals to promote behaviour change, or reach those who may need support. Understanding its effectiveness will provide important learning on how public health information can be communicated to people more broadly.

This study aimed to evaluate the #TimeToBeKind campaign film. The primary hypothesis was that watching the #TimeToBeKind campaign film would result in increased intention to act in ways which promote kindness to others and, consequently, a greater likelihood that members of the public would provide support to vulnerable individuals or others in need. Further hypotheses were that individuals would be more likely to report behavioural intentions for kindness if the film provoked an emotional reaction (e.g. if the film made them feel upset or sad, hopeful or encouraged) and if the film resonated with their experience of COVID-19 lockdown restrictions. The #TimeToBeKind campaign and materials were developed by the Welsh ACE Support Hub and Cowshed Communication. The evaluation team had no role in the design or development of any part of the campaign or its materials.

## Methods

A mixed methods approach was undertaken using: a short online survey with a convenience sample to explore public attitudes towards the film and behavioural intentions for kindness; and analysis of social media interaction with the film on Twitter (a platform that enables users to create and share a ‘tweet’ of up to 280 characters), to explore attitudes towards the film and examples of behaviour change.

### Online survey

The online survey used a five point Likert scale (1 = strongly disagree, 5 = strongly agree) to capture: participant opinions on the film (i.e. ease of understanding, contained useful information, understood campaign message and credibility); how the film made the participants feel (i.e. upset or sad, hopeful or encouraged, difficult or distressing, gained something positive, glad watched, important issues talked about, resonated with lockdown experience); their own kindness and compassion (kind to others, feel sorry for others, try to be caring, help others out); and behavioural intentions for kindness as a result of viewing the film (to be kind to others, to check in on friends and family, keep an eye out for people experiencing these issues, awareness and confidence in intervening in situations). Participants were also asked: where they first saw the film; their prior knowledge of the ACE Support Hub; to rate the amount of information in the film and its length; to rate how memorable the film was (1 = not at all, 10 = extremely); and demographic information (age [5 year age groups], gender [male, female, other], employment status, profession, area of residence [England, Scotland, Northern Ireland, Welsh Unitary Authority level, other]). Participants were able to leave additional feedback on the campaign film, collected using a free-text box. All outcomes were self-reported.

The online survey (hosted on Survey Monkey) was embedded on the #TimeToBeKind pages of the ACE Support Hub website (www.aceawarewales.com). Social media posts (Facebook, Instagram and Twitter platforms) in conjunction with social media campaign coverage prompted individuals to complete the survey. An information sheet outlining: the study aims; its voluntary, confidential and anonymous nature; and arrangements for data use and storage, was made available to all potential participants. Participants provided active consent (recorded online) to participate. Following completion of the questionnaire, information was provided on a range of organisations and support services. Individuals aged 16 years or over were eligible to participate, and English and Welsh language versions of the information sheet, study questionnaire and support services were available. Based on significance levels from previous studies [26], a minimum sample size of 277 survey responses was set to enable suitable statistical power for analysis.

Data collection was completed online during the #TimeToBeKind campaign period plus an additional week (until 4th May 2021) and was completed by 399 individuals (85.7% in English, 14.3% in Welsh). Due to missing demographic data, nine responses were excluded, leaving a final sample for analysis of 390.

### Twitter

The Twitter advanced search engine function was searched for publicly available tweets using the hashtag #TimeToBeKind and its Welsh language translation #AmserifodYnGaredig. Search parameters were set to capture tweets posted between the seven-week evaluation period, with no limitation on language or geography. Returned tweets were manually entered into Excel, recording: content, time and date, tweet type (i.e. original tweet or re-tweet/quote tweet [where a user retweets another user’s content/adds their own comment]), number of re-tweets/quote tweets, ‘likes’ and comments. User comments and replies on returned tweets were also extracted. Where available, information was collected from the user profile, including: name, biography, geographical location and number of tweets and followers.

The search retrieved 163 tweets. Welsh language tweets were translated to English. Tweets which were not of relevance to the campaign (*n* = 8), and those which advertised the online survey (*n* = 11) were excluded. Tweets by the film’s publisher (including those employed in the same organisation, *n* = 29), and stakeholders (*n* = 58) which used content provided by the ACE Support Hub, were excluded due to bias. All tweets were categorised for inclusion and exclusion by two reviewers (KF and RH). There was an excellent level of agreement in coding between reviewers (95.7%), Cohen’s κ 0.904. Following screening, a final sample of 57 tweets were available for analysis.

### Analysis

Online survey data was cleaned, with age coded into groups (16-29, 30-39, 40-49 and 50+ years) and Likert scale responses dichotomised into agree (strongly agree/agree) versus disagree/neither (neither agree/disagree, disagree, strongly disagree). Employment was coded into employed, student, other (unemployed, those not working for domestic reasons, long-term sick or disabled, retired and carers). Profession was grouped into health or social services, employed in another public service (e.g. police, education) and other (employed in another sector, student, unemployed and retired). Descriptive statistics explored the demographics of the sample, attitudes towards the film, how it made the participant feel, and behavioural intentions for kindness, with bivariate analysis employed to analyse any association between demographics (gender, age), how the film made them feel and behavioural intentions for kindness using SPSS v.25. Free-text feedback was analysed thematically using NVIVO.

Tweets were coded for their sentiment (positive, negative, or neutral) towards the campaign using guidelines for best practice [33–35]. Tweets that indicated emotions such as sadness were coded as negative and tweets that were simultaneously negative and positive were coded as neutral. Nine tweets identified acts of kindness (behaviour change) as a result of the campaign, but offered no sentiment on the campaign itself, thus were not sentiment rated. Sentiment coding between reviewers (KF and RH) produced 94.7% agreement, Cohen’s κ 0.857. Using the profile description, twitter users were categorised as an individual user or an organisation. Analysis was run in SPSS v.25.

### Ethics

 The Bangor University School of Health Sciences and Medical Sciences Ethical Committee provided ethical approval for the study (reference: 2021-16896). Twitter data collection and analysis followed guidelines on the use of Twitter data for data collection and analysis [36], thus tweets are not presented at the individual level here.

## Results

### Online survey

The demographic breakdown of participants is shown in Table 1. The majority of participants were female (81.5%), aged 40+ (55.9%) and resident in Wales (89.2%). Over seven in ten (71.0%), were currently employed, of whom a third (32.8%) worked in health or social services, a quarter (25.0%) in other public sector roles, over one in five (22.4%) in educational or childcare settings, and 6.7% in the charity/voluntary sector. Almost seven in ten respondents (68.6%) were not aware of the ACE Support Hub before they viewed the campaign film and 72.9% completed the survey the same day they first saw the film. One in four (39.5%) respondents first viewed the film on social media, with under one in five (18.7%) having first seen the film on television.

**Table 1:** Survey participant demographics and film viewing

		n	%
**All**		390	
**Sex**	Male	72	18.5
	Female	318	81.5
**Age (years)**	16-29	100	25.6
	30-39	72	18.5
	40-49	95	24.4
	50+	123	31.5
**Employment**	Employed	277	71.0
	Student	65	16.7
	Other^a^	48	12.3
**Profession**	Health or social services	88	32.8
	Education or childcare	60	22.4
	Other public sector^b^	67	25.0
	Charity/voluntary	18	6.7
	Other^c^	35	13.1
**Country of residence**	Wales	348	89.2
	England	35	9.0
	Other^d^	7	1.8
**When did you first see the film (*n*** **= 387)**	Today	282	72.9
	In the last week	54	14.0
	>1 week ago	51	13.2
**Where did you first see the film**	TV	73	18.7
	Social media	154	39.5
	ACE Support Hub website	16	4.1
	Friend/family/colleague shared it	89	22.8
	Link from newsletter/other website	49	12.6
	Other (including search engine, don't remember)	9	2.3
**Aware of the ACE Support Hub before saw the film (*n*** **= 389)**	Yes, aware	122	31.4
	No, not aware	267	68.6

*ACE* Adverse childhood experience. ^a^Those unemployed, long-term sick and disabled, retired, carer, and those not working for domestic reasons. ^b^Local or national government and police or criminal justice. ^c^Private sector, hospitality and beauty industries. ^d^ Includes not reported

Participant attitudes towards the film were positive. The majority reported that the length of the film and the amount of information in the film was *just right* (82.5 and 73.3% respectively; Table 2). The majority agreed the film was easy to understand (94.4%); they understood the campaign message (91.8%); the film contained useful information (75.4%); they trusted that the film was from a credible source (88.5%); and that it was memorable (89.4%). Despite over two thirds (67.9%) agreeing that watching the film made them feel upset or sad and a third (34.6%) finding the film difficult or distressing to watch, over half (55.9%) reported that the film left them feeling hopeful or encouraged. The majority agreed that: they gained something positive from watching the film (72.8%), were glad they watched it (82.1%) and that it is important that the issues in the film are talked about (98.2%, Table 2). One in five (22.6%) agreed that the film resonated with their experience of lockdown.

**Table 2:** Survey participant attitudes towards the film and reported kindness and behavioural change^a^

	% reporting		
**Perceptions of the film**	**Too little**	**Just right**	**Too much**
The amount of information in the film was	25.4	73.3	1.3
	**Too short**	**Just right**	**Too long**
The length of the film was (*n* = 389)	15.9	82.5	1.5
	**Disagree** ^b^ **/ neither**		**Agree** ^c^
The film was easy to understand	5.6		94.4
The film contained useful information	24.6		75.4
I understand the campaign message	8.2		91.8
I trust that the film is from a credible source	11.5		88.5
**Reactions to the film**			
Watching the film made me feel upset or sad	32.1		67.9
Watching the film made me feel hopeful or encouraged	44.1		55.9
The film was difficult or distressing to watch	65.4		34.6
I gained something positive from watching the film	27.2		72.8
I am glad I watched the film	17.9		82.1
I think it is important that the issues in the film are talked about	1.8		98.2
The film resonated with my experience of being in lockdown/My experiences of lockdown have been similar to those in the film	77.4		22.6
**Own kindness**			
I am kind to others	3.6		96.4
I feel sorry for other people when they experience problems	4.1		95.9
If I see someone going through a difficult time, I try to be caring towards that person	2.6		97.4
I like helping other people out, even strangers	8.2		91.8
**Outcomes/behaviour change**			
I am more likely to be kinder to others	36.4		63.6
I am more likely to check in on friends, family and neighbours	29.5		70.5
I am more likely to keep an eye out for friends and family experiencing situations like those in the film	19.5		80.5
I am more likely to try to help other members of my community	34.4		65.6
I am more aware of situations like those highlighted by the film	29.5		70.5
I feel more confident that I could intervene in situations like those highlighted in it, if I wanted to	39.5		60.5
The film has made me more likely to intervene in situations like those highlighted	36.2		63.8
**Memorable**	**scores 0-5**		**scores 6-10**
On a scale of 1 to 10, where 1 is not at all and 10 is extremely, how memorable do you think the film is? (*n* = 386)	10.6		89.4

^﻿a^Unless otherwise reported uses full sample. ^b^Includes strongly disagree. ^c^Includes strongly agree

Despite participants reporting high levels of kindness (see Table 2), overwhelmingly participants reported that after watching the film they had behaviour intentions for kindness. Across the intention to change outcomes, the majority agreed that as a result of seeing the film they would be more likely to: be kinder to others (63.6%), try and help other members of their community (65.6%), and check in on friends, family and neighbours (70.5%). Eight in ten (80.5%) agreed that as a result of seeing the film they were more likely to keep an eye out for friends and family experiencing situations like those in the film. The majority of respondents also agreed that as a result of seeing the film they were more aware of situations like those highlighted by the film (70.5%), felt more confident that they could intervene in situations like those highlighted in the film (60.5%) and that the film made them more likely to intervene in situations like those highlighted (63.8%).

**Table 3:** Percentage reporting behaviour intentions by demographics, emotional reaction to the film and resonation with lockdown experience

	I am more likely to be kinder to others				I am more likely to check in on friends, family and neighbours				I am more likely to keep an eye out for friends and family experiencing situations like those in the film				I am more likely to try to help other members of my community			
	Disagree/ neither	Agree	*X* ^2^	p	Disagree/ neither	Agree	*X* ^2^	p	Disagree/ neither	Agree	*X* ^2^	p	Disagree/neither	Agree	*X* ^2^	p
**Gender**																
Male	48.6	51.4			34.7	65.3			29.2	70.8			47.2	52.8		
Female	33.6	66.4	5.677	0.017	28.3	71.7	1.164	0.281	17.3	82.7	5.273	0.022	31.4	68.6	6.478	0.011
**Age (years)**																
16-29	33.0	67.0			25.0	75.0			14.0	86.0			31.0	69.0		
30-39	37.5	62.5			31.9	68.1			16.7	83.3			43.1	56.9		
40-49	29.5	70.5			26.3	73.7			16.8	83.2			26.3	73.7		
50+	43.9	56.1	5.496	0.139	34.1	65.9	2.921	0.404	27.6	72.4	7.921	0.048	38.2	61.8	6.449	0.092
**Watching the film made me feel upset or sad**																
Disagree/neither	48.8	51.2			42.4	57.6			29.6	70.4			42.4	57.6		
Agree	30.6	69.4	12.197	<0.001	23.4	76.6	14.753	<0.001	14.7	85.3	11.991	0.001	30.6	69.4	5.270	0.022
**Watching the film made me feel hopeful or encouraged**																
Disagree/neither	55.2	44.8			45.3	54.7			32.0	68.0			50.6	49.4		
Agree	21.6	78.4	47.084	<0.001	17.0	83.0	37.233	<0.001	9.6	90.4	30.593	<0.001	21.6	78.4	35.905	<0.001
**The film was difficult or distressing to watch**																
Disagree/neither	36.9	63.1			30.6	69.4			20.4	79.6			35.3	64.7		
Agree	25.6	64.4	0.065	0.799	27.4	72.6	0.430	0.512	17.8	82.2	0.385	0.535	32.6	67.4	0.286	0.593
**I gained something positive from watching the film**																
Disagree/neither	66.0	34.0			57.5	42.5			45.3	54.7			63.2	36.8		
Agree	25.4	74.6	55.186	<0.001	19.0	81.0	55.122	<0.001	9.9	90.1	61.736	<0.001	23.6	76.4	53.714	<0.001
**I am glad I watched the film**																
Disagree/neither	80.0	20.0			71.4	28.6			55.7	44.3			77.1	22.9		
Agree	26.9	73.1	70.012	<0.001	20.3	79.7	72.177	<0.001	11.6	88.4	71.362	<0.001	25.0	75.0	69.240	<0.001
**I think it is important that the issues in the film are talked about**																
Disagree/neither	100.0	0.0			85.7	14.3			71.4	28.6			100.0	0.0		
Agree	35.2	64.8	12.449	<0.001	28.5	71.5	10.838	0.001	18.5	81.5	12.257	<0.001	33.2	66.8	13.618	<0.001
**The film resonated with my experience of being in lockdown**																
Disagree/neither	39.4	60.6			32.5	67.5			23.5	76.5			39.1	60.9		
Agree	26.1	73.9	5.181	0.023	19.3	80.7	5.652	0.017	5.7	94.3	13.805	<0.001	18.2	81.8	13.186	<0.001

**Table 4:** Percentage reporting behaviour intentions by demographics, emotional reaction to the film and resonation with lockdown experience

	I am more aware of situations like those highlighted by the film				I feel more confident that I could intervene in situations like those highlighted in it, if I wanted to				The film has made me more likely to intervene in situations like those highlighted			
	Disagree/neither	Agree	*X* ^2^	*p*	Disagree/neither	Agree	*X* ^2^	*p*	Disagree/neither	Agree	*X* ^2^	*p*
**Gender**												
Male	36.1	63.9			51.4	48.6			44.4	55.6		
Female	28.0	72.0	1.863	0.172	36.8	63.2	5.235	0.022	34.3	65.7	2.629	0.105
**Age (years)**												
16-29	24.0	76.0			34.0	66.0			31.0	69.0		
30-39	30.6	69.4			43.1	56.9			44.4	55.6		
40-49	25.3	74.7			34.7	65.3			27.4	72.6		
50+	36.6	63.4	5.283	0.152	45.5	54.5	4.420	0.220	42.3	57.7	8.469	0.037
**Watching the film made me feel upset or sad**												
Disagree/neither	46.4	53.6			54.4	45.6			52.0	48.0		
Agree	21.5	78.5	25.308	<0.001	32.5	67.5	17.122	<0.001	28.7	71.3	20.012	<0.001
**Watching the film made me feel hopeful or encouraged**												
Disagree/neither	43.6	56.4			59.9	40.1			53.5	46.5		
Agree	18.3	81.7	29.495	<0.001	23.4	76.6	53.573	<0.001	22.5	77.5	40.056	<0.001
**The film was difficult or distressing to watch**												
Disagree/neither	31.4	68.6			43.1	56.9			38.4	61.6		
Agree	25.9	74.1	1.259	0.262	32.6	67.4	4.107	0.043	31.9	68.1	1.655	0.198
**I gained something positive from watching the film**												
Disagree/neither	55.7	44.3			61.3	38.7			59.4	40.6		
Agree	19.7	80.3	47.958	<0.001	31.3	68.7	29.040	<0.001	27.5	72.5	34.177	<0.001
**I am glad I watched the film**												
Disagree/neither	68.6	31.4			80.0	20.0			74.3	25.7		
Agree	20.9	79.1	62.678	<0.001	30.6	69.4	58.600	<0.001	27.8	72.2	53.740	<0.001
**I think it is important that the issues in the film are talked about**												
Disagree/neither	85.7	14.3			100.0				100.0			
Agree	28.5	71.5	10.838	0.001	38.4	61.6	10.923	0.001	35.0	65.0	12.588	<0.001
**The film resonated with my experience of being in lockdown**												
Disagree/neither	33.1	66.9			44.0	56.0			39.7	60.3		
Agree	17.0	83.0	8.461	0.004	23.9	76.1	11.609	0.001	23.9	76.1	7.437	0.006

Relationships between behaviour intentions for kindness as a result of seeing the film and participant demographics are shown in Tables 3 and 4. A significantly higher proportion of those who had an emotional reaction to the film (vs. those not emotionally affected) agreed they were more likely to adopt kindness behaviours as a result of seeing the film (Tables 3 and 4). Thus, a higher proportion of those who agreed the film made them feel upset or sad (vs. those not sad or upset) also agreed that the film made them: more likely to be kinder to others (69.4% of those upset or sad vs. 51.2% of those not upset or sad; *p* < 0.001; Table 3), more aware of situations like those highlighted by the film (78.5% vs. 53.6%; *p* < 0.001; Table 4); feel more confident to intervene in situations like those highlighted (67.5% vs. 45.6%; *p* < 0.001; Table 4); and more likely to intervene in such situations (71.3% vs. 48.0%; *p* < 0.001; Table 4). A similar significant relationship was found for all behavioural intentions for kindness outcomes and those who agreed with the statements (vs. those who disagreed): Watching the film made me feel hopeful or encouraged, I gained something positive from watching the film, and I think it is important that the issues in the film are talked about.

There was no significant association between behavioural intention outcomes and whether respondents found the film difficult or distressing to watch, except for the statement I feel more confident that I could intervene in situations like those highlighted in it, with a higher proportion of those finding the film difficult/distressing reporting the intention than those who did not. A higher proportion of those reporting they found the film difficult/distressing to watch (vs. those that did not) reported that as a result of seeing the film they felt confident to intervene in situations like those highlighted in it (67.4% agree difficult distressing, 56.9% disagree difficult/distressing, *p* = 0.043; Table 4).

A higher proportion of those who agreed that the film resonated with their experience of being in lockdown (vs. those that did not) also agreed with all behaviour intentions for kindness as a result of seeing the film: 81.8%, agreed as a result of seeing the film I am more likely to try to help other members of my community, vs. 60.9% of those who reported the film did not resonate with their experience (*p* < 0.001; Table 3).

#### Online survey qualitative feedback

Free-text feedback on the campaign film was left by 105 survey respondents. Of these, qualitative analysis highlighted that six respondents had a poor understanding of the campaign message and did not understand what action they were intended to follow post viewing:

There wasn’t a clear message or action in the video so unsure what I am supposed to do with the information.

Not sure what the message of the film was, offer support?

Forty-five comments proposed recommended changes to the video, predominantly for the inclusion of additional information as to where individuals can get help and support, both to cope with their own experience of adversity but also for advice on how to help others who may be suffering. This included additional information such as where to go for ‘immediate help’ or ‘safeguarding’ concerns. Other common themes included that the film was too short in length, and that its speed made it difficult for the viewer to read the text:

It is easy to miss the words to read at the end, especially as they are only there for a short time.

I did feel it tried to show too much in the short amount of time.

Across the responses, thirteen comments indicated that the film had evoked a strong emotional reaction. Responses commonly described the film as ‘powerful’ and having made participants ‘emotional’. However, despite feeling emotionally affected, respondents reported that they viewed the film positively and that it had an affirmative effect on them:

It made me cry, because I thought of all the children who are going through difficult times. It felt encouraged that I can help others.

I thought it was very powerful - it made me cry. It seemed very real and raw.

I thought it was really good but a little upsetting but feel that the upset is what would make people sit up and listen.

Nine individuals indicated their own experience of childhood adversity, but the impact of the film on them varied. One individual felt that the film was ‘distressing’ and one noted that they felt ‘a content warning could be necessary’. However, others who had reported personal experiences of adversity reported that they thought that it was important that these messages are shared, and hoped that campaigns such as this would raise awareness and evoke change:

Absolutely brilliant to see this on television. I was abused as a child and raising awareness of abuse and difficult home life is so important. Had this been on the television when I was young my life may have been very different. People are now being educated to intervene and check on each other which is absolutely brilliant and will save lives.

Overall feedback was mostly positive (46 comments), highlighting that the ‘film was interesting and thought provoking’ and that it would ‘resonate with many’. Others also noted that they thought the film was a good start to an education on ACEs and that they felt encouraged by the film:

It was a brilliant advert for a difficult subject. Thank you.

I think it was a really good and effective clip and really captured the message well.

Seven individuals also reported that additional information on what ACEs are and what the ACE Support Hub do would be useful, and that future videos highlighting other ACE types should be created.

### Twitter data

#### Source

The 57 included tweets were produced by 49 unique Twitter users (Table 5). The majority of accounts only had one included tweet (mean 1.2). The number of followers for each user account ranged from 1->5,000. Of users who could be assigned a geography (*n* = 44), 61.4% (*n* = 27) were located in Wales. Over two thirds (65.3%) of accounts were categorised as individual users.

Across all tweets, the majority (86.0%) had been ‘liked’ by other Twitter users, receiving a total of 234 likes (range 1-16). Half of all tweets (50.9%) had been re-tweeted/quote-tweeted, totalling 54 times (range 1-6). Over one in ten tweets (15.8%, *n* = 9) received a comment by another Twitter user, of which 7 received one comment, and two received two comments.

**Table 5:** Twitter user demographics, sentiment and tweet interaction

			N	%
**Users (*n*** **= 49)**				
**Mean number of tweets per user** (Range)			1.2 (1-4)	
**User geography**	Wales		27	55.1
	Other^a^		17	34.7
	Unidentifiable		5	10.2
**User source**	Individual		32	65.3
	Organisation		17	34.7
**All tweets**				
**Tweet sentiment (*n*** **= 48)**	Positive		33	68.8
	Neutral		11	22.9
	Negative		4	8.3
**Interaction with tweet (*n*** **= 57)**	Liked		49	86.0
	Re-tweeted/quote-tweeted		29	50.9
	Commented on		9	15.8

^a^Includes UK wide, England, Scotland, Caribbean, Canada, USA

#### Tweet sentiment

Forty-eight tweets were sentiment rated. The majority (*n* = 33, 68.8%; Table 5) were coded as expressing a positive sentiment towards the film, 11 neutral (22.9%; predominately shared the campaign hashtag/film link) and four (8.3%) negative. There was no association between sentiment versus user source (*p* = 0.317). There was an association between tweets being ‘liked’ and tweet sentiment, with 93.9% of tweets rated positive ‘liked’, 81.8% of those rated neutral ‘liked’ and no tweets which were rated as negative ‘liked’ (*p* < 0.001). However, there was no association between sentiment and being re-tweeted/quote-tweeted (*p* = 0.117), or commented on (*p* = 0.745).

## Discussion

This research provides an understanding of the public response to a short campaign film promoting behaviour change for kindness during a period of COVID-19 restrictions. Findings from the online survey indicated a positive public reaction to the film and good understanding of the campaign message, with only six respondents providing free-text feedback indicating poor understanding of the campaign message. Moreover, the majority of interaction with the film on the social media platform Twitter expressed positive sentiment, of which over nine in ten tweets were ‘liked’ by other users.

 Study findings confirmed the primary hypothesis of the evaluation - that watching the #TimeToBeKind campaign film would result in participants reporting behaviour intentions for acts of kindness to others. The majority of participants agreed that as a result of seeing the film they would be more likely to: be kinder to others, try and help other members of their community, and keep an eye out for friends and family experiencing situations like those depicted in the film. These findings indicate the potential for public information films to influence behaviour intentions, aligning with previous research showing that interventions which are persuasive in nature can help to reinforce beneficial behaviours [37].

Even without specialist training, individuals can help other members of society by completing acts of kindness. These natural acts and non-specialist people skills are beneficial to increasing resilience, which could combat the negative outcomes associated with experiencing ACEs. Research has shown that having continuous trusted adult support in childhood and feeling supported and culturally connected in adulthood goes some way to build resilience to prevent the negative outcomes associated with ACEs [18, 38, 39]. Such acts of kindness can also benefit the individual committing the act, as research has shown that engagement in positive activities can reduce negative emotions, increase happiness and positively impact mental health [37, 40, 41].

It is evident that for many respondents, viewing the film evoked a strong emotional reaction. Despite over two thirds of online respondents reporting that the film made them feel upset or sad, the majority also reported that they had gained something positive from watching the film. Importantly, being emotionally affected by the film (e.g. feeling upset or sad) was shown to increase reporting of intentions to adopt kindness behaviours, and to intervene in situations like those depicted. Free-text feedback supported this, with respondents viewing the film positively despite feeling ‘emotional’. Findings indicate that films can evoke strong emotions such as sadness, and that this emotional arousal can encourage individuals to make positive behaviour change. These results contribute to other research which has identified that the evocation of an emotive response to public information films is not a negative thing [26]. The emotional tone of messaging can influence its effectiveness. Here, the evocation of sadness may have improved public acceptance of the film’s message. Employing emotion is commonly used in advertising by charitable organisations to invoke sympathy and encourage donations [42]. Research has also shown that messages which arouse strong negative emotions (e.g. sadness) are subject to better recall [43, 44]. However, we were unable to measure recall in the present study. These findings around emotional arousal are important for our broader understanding of public health messaging. It is imperative to recognise that individuals can feel that they have benefitted from viewing content that upsets them. In this sense, being emotionally affected by public health films may be no different to having an emotional reaction to fictional or non-fictional video content and this emotional response may be important for promoting behaviour change.

Individuals who reported personal experiences of adversity in free-text feedback, also indicated that they thought the campaign was important and that awareness should be raised for such issues. These findings echo those of an evaluation of a short film communicating ACEs to the public, which identified that despite being emotionally affected by the film, individuals who had experienced ACEs also felt people would benefit from watching the film [26]. Results here also indicated that individuals whose experiences of lockdown were similar to those depicted in the film were more likely to report intentions for kindness as a result of watching the film. This finding is not unexpected given that the experience of adversity can lead to increased empathy and compassion for others [45]. Demonstrating that public health information films can encourage kindness behaviour change is of relevance to other public health issues and for future pandemics which may see a return to social restrictions similar to those imposed in response to COVID-19.

Despite being created in response to concerns about increased ACE exposure as a result of COVID-19, learning from this evaluation can be utilised more broadly for public health information films. The findings evidence that public health messaging can use innovative methods to reach people, including those who are isolated. In the present study, one in four (39.5%) respondents first saw the film on social media, with under one in five (18.7%) having first seen the film on television. With increasing time being spent online and short video content becoming progressively popular through social media platforms such as YouTube, Instagram and TikTok, it is important that the potential of online short films/videos as a public health tool is recognised. COVID-19 has accelerated the move to an online presence for many individuals, thus, the importance of this space for public health messaging and communication has grown. Social media has been used to explore public attitudes towards health interventions [33, 34, 46] yet only a small body of research identifies the influence of social media interventions on intentions to change health behaviour, as few online interventions are fully evaluated [47]. However, the impact of such work needs to be fully understood to ensure public health messaging is effective.

Almost seven in ten survey respondents (68.6%) reported they had not been aware of the Welsh ACE Support Hub before they saw the #TimeToBeKind campaign, thus identifying the film’s reach to a new audience. Generally multi-media approaches are not targeted, but as television viewing figures for the film evidence [23], do reach a large audience. In 2020, use of digital online video, streaming or subscription services dramatically increased [48], particularly amongst youth [49], which is in line with the majority of survey respondents reporting they viewed the #TimeToBeKind film online. However, the reach and economics of a television and online public health messaging approach as opposed to more targeted approaches need to be further explored. Here, males and youths were underrepresented in the online sample yet were target groups for the campaign film, and are increasingly being targeted to encourage communication with others on their mental health and wellbeing (for example the Mental Health Foundation #UnlockLoneliness campaign, https://www.mentalhealth.org.uk/campaigns/unlock-loneliness). However, we were unable to identify the demographics of those who watched the film, something which future work should try to capture. Thus, further work is needed to explore the positioning and reach of online public health information films in comparison to more targeted approaches, and the appropriate research methodologies that can evaluate their effectiveness.

Feedback from the survey indicated potential changes that future public health information films should consider. Firstly, there is a need to provide clear messages on what actions people should take following viewing such films. Some respondents did not understand what action they were intended to follow post-viewing and thought the film required additional information on help and support services. The addition of such information is vital, especially for individuals who may currently be exposed to trauma such as ACEs as portrayed in the film. Secondly, attention needs to be paid toward ensuring that the pace and timing of films are balanced to enable all viewers, including those with low literacy levels, the ability to absorb the campaign message. Future public health films should consider embedding research/evaluation into the film production process to ensure that there is good understanding of film messages and that such films do not have unintended consequences.

There are a number of limitations to this study. The #TimeToBeKind campaign did not provide a definition for kindness. Consequently the evaluation materials did not define kindness, and the meaning of kindness, or what respondents understood kindness or compassion to be was not directly measured. We recognise that individuals may hold different interpretations for kindness and what it means to be kind. Future research should explore the concepts of kindness and their meaning further with the general public. Due to the moderate survey sample size there is limited generalisability of the study findings. In particular, males and youths were under-represented, and these may be two key target groups for such interventions. We were unable to identify any selective bias created by non-participation and we cannot guarantee that respondents viewed the whole film. However, a strength of the evaluation is the use of mixed-methods with varied samples to explore attitudes towards the film and resulting intentions for behaviour change. The survey did not include any direct measures of participants’ change in behaviour, instead measuring intentions for change. Due to the anonymous nature of the survey we were unable to explore if the film’s evocation of sadness led to increased long-term memorability, recall or change in practice, which future research should consider exploring.

A small number of users interacted with the film on Twitter. Although the majority of users were based in Wales where the film was broadcast, the sample is not indicative of all viewers. Twitter data were collected using the Twitter search interface, a facility that is limited to retrieving tweets from publicly available accounts that had posted a tweet using the campaign hashtag, or those commenting on such tweets. Captured tweets therefore relate to a small number of users who are unrepresentative of the general population. Twitter accounts also provided limited biographical information, preventing further analysis by demographics.

## Conclusions

The results of this evaluation show positive attitudes amongst members of the public to the #TimeToBeKind film and intentions for behaviour change – to make acts of kindness to others - as a result of watching the film. The findings demonstrate how film can be an effective tool to promote behaviour change for kindness. Furthermore, the study suggests that films which evoke arousal of emotional reactions such as sadness, can still be perceived positively by viewers and can encourage individuals to make positive behaviour change. The learning from this evaluation is of use to the future development of public health information films that seek to instil behaviour change. The findings evidence that public health messaging can use innovative methods such as film to reach individuals, including those who are isolated. With the COVID-19 pandemic accelerating a move to working and communicating online for many, the findings of the present evaluation are also relevant to how public health messaging can adapt and utilise online space to target individuals and promote behaviour change.

## Data Availability

The datasets used and/or analysed during the current study are not publicly available due to limitations of ethical approval but are available from the corresponding author on reasonable request.

## Abbreviation

ACE: Adverse childhood experience
